# Supplementary material for: A Real-World Cost-Effectiveness Study Evaluating Imaging Strategies for the Diagnostic Workup of Renal Colic in the Emergency Department
Source: Medicina (Kaunas). 2023 Feb 28;59(3):475. doi: 10.3390/medicina59030475 (PMC10058030; doi:10.3390/medicina59030475)
Supplement: Supplementary file 1 [file medicina-59-00475-s001.zip › medicina-2190111-supplementary.pdf]

Supplementary material

Table S1. Unit costs of resources use in euros.

	**Unit Cost**	**source**
Direct healthcare costs (national estimates)		
Consultation in the ED	€80	
US	€62.5	*Social health insurance schedule*
CT	€78.5	
Hospital admissions (French diagnosis related group)		
Urinary stone, very short time n = 217 (79.6%)	€673	National cost study
Transuretral or transcutaneous intervention for urinary stone n = 56 (20.4%)	€2139	

* Weighted average for all four severity levels; ED emergency department; CT computed tomography; US ultra
